# Population Structure of the Endangered Franciscana Dolphin (*Pontoporia blainvillei*): Reassessing Management Units

**DOI:** 10.1371/journal.pone.0085633

**Published:** 2014-01-31

**Authors:** Haydée A. Cunha, Bruna V. Medeiros, Lupércio A. Barbosa, Marta J. Cremer, Juliana Marigo, José Lailson-Brito, Alexandre F. Azevedo, Antonio M. Solé-Cava

**Affiliations:** 1 Laboratório de Biodiversidade Molecular, Instituto de Biologia, Universidade Federal do Rio de Janeiro, Rio de Janeiro, Brazil; 2 Laboratório de Mamíferos Aquáticos e Bioindicadores, Faculdade de Oceanografia, Universidade do Estado do Rio de Janeiro, Rio de Janeiro, Brazil; 3 Organização Consciência Ambiental, Vila Velha, Espírito Santo, Brazil; 4 Departamento de Ciências Biológicas, Universidade da Região de Joinville, Joinville, Santa Catarina, Brazil; 5 Laboratório de Patologia Comparada de Animais Selvagens, Faculdade de Medicina Veterinária e Zootecnia, Universidade de São Paulo, São Paulo, Brazil; 6 Projeto Biopesca, Praia Grande, São Paulo, Brazil; University of Illinois at Urbana-Champaign, United States of America

## Abstract

Franciscanas are the most endangered dolphins in the Southwestern Atlantic. Due to their coastal and estuarine habits, franciscanas suffer from extensive fisheries bycatch, as well as from habitat loss and degradation. Four Franciscana Management Areas (FMA), proposed based on biology, demography, morphology and genetic data, were incorporated into management planning and in the delineation of research efforts. We re-evaluated that proposal through the analysis of control region sequences from franciscanas throughout their distribution range (N = 162), including novel sequences from the northern limit of the species and two other previously unsampled localities in Brazil. A deep evolutionary break was observed between franciscanas from the northern and southern portions of the species distribution, indicating that they must be managed as two Evolutionarily Significant Units (ESU). Furthermore, additional FMAs should be recognised to accommodate the genetic differentiation found in each ESU. These results have immediate consequences for the conservation and management of this endangered species.

## Introduction

The franciscana *Pontoporia blainvillei* (Gervais & d'Orbigny, 1844), is a small dolphin endemic to the Southwestern Atlantic, from the state of Espírito Santo, Brazil (∼18°S), to the province of Chubut, Argentina (∼42°S) [1]. It belongs to a relict lineage and its closest living relative is the riverine boto, *Inia geoffrensis* [2], [3] which occurs in the Amazon and Orinoco river basins.

Franciscanas are the most endangered dolphins in the Southwestern Atlantic [4], [5] representing the only South Atlantic dolphin species in the Red List of the International Union for Conservation of Nature (listed as vulnerable, A3d). Due to their coastal and estuarine habits, franciscanas inhabit areas of heavy human activity, which poses several threats to their conservation. For example, franciscanas are the most frequent cetacean species in incidental captures along most of their range [6], [7], [8], [9], [10], [11], [12], [13], and, where basic data have been gathered, current levels of bycatch have been shown to be unsustainable [14], [15], resulting, in southern Brazil, in a population decrease of more than 30% projected over three generations [14], [16], [17]. Habitat loss and degradation are other major threats, as much of the species' habitat has been or is expected to be modified in the near future. Where franciscanas still exist in proximity to urban centers, contamination levels are also a matter of concern [18], [19], [20], [21], [22], [23].

To help the conservation of *Pontoporia* populations, it is fundamental that their limits be clearly identified. Delimitation is vital to access demographic parameters and, thus, the impact of non-natural mortality. Being demographically independent, populations need to be managed separately. Genetic data have the unrivalled ability to disclose demographically independent units. In conservation, those units are called Evolutionarily Significant Units [24] or Management Units (*sensu* Moritz [25]), depending on the degree of evolutionary divergence among them.

**Figure 1:**
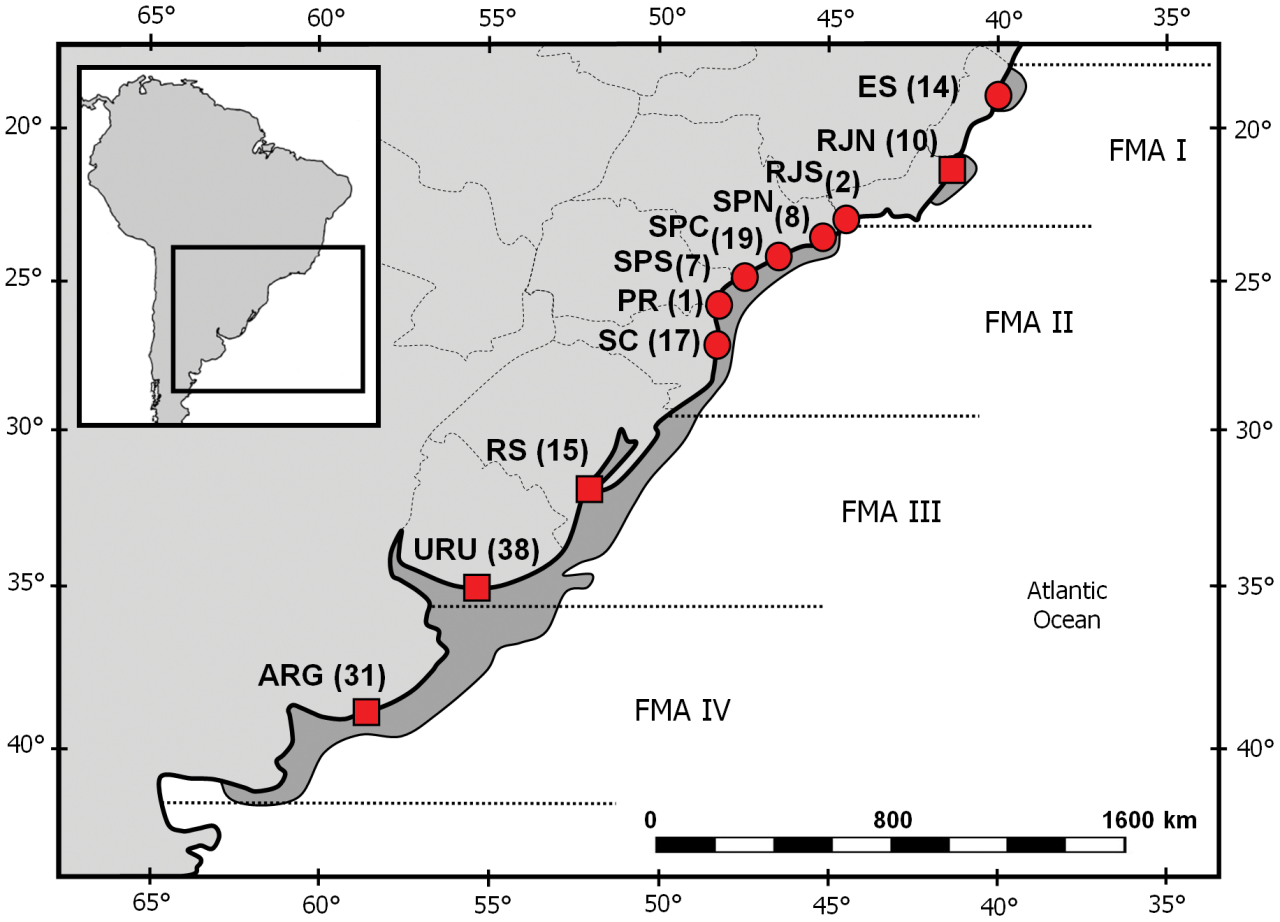
Franciscana Management Areas (FMA) and sampling. Sample sizes and localities across the species' distribution (dark grey) and the four FMAs (I to IV) proposed by Secchi et al. (2003). Circles indicate new samples, squares indicate sequences from the literature (Secchi et al. 1998, Lázaro et al. 2004). ES: Espírito Santo; RJN: northern Rio de Janeiro; RJS: southern Rio de Janeiro; SPN: northern São Paulo; SPC: central São Paulo; SPS: southern São Paulo; PR: Paraná; SC: Santa Catarina; RS: Rio Grande do Sul; URU: Uruguay; ARG: Argentina.

Secchi et al. [26] compiled all available information at the time, including genetic data, and proposed four Franciscana Management Areas (FMA, Fig. 1). The FMA were incorporated into management planning and in the delineation of subsequent research efforts. Since then, more genetic data have accumulated in favour of that proposal [27], [28], but recent studies have also argued for finer subdivision within the two southern FMA (III and IV) [28], [29], [30]. However, none of those studies included samples from the northernmost region of the species distribution, in the state of Espírito Santo. In this study, we analysed control region sequences from franciscanas throughout the species distribution range, including novel sequences from three localities previously unsampled (Espírito Santo, southern Rio de Janeiro and northern Santa Catarina). Our results reformulate the proposal of Secchi et al. [26] and have immediate consequences for the conservation and management of the species.

## Materials and Methods

We collected samples from 68 franciscana carcasses that had washed ashore along the Brazilian coast (Fig. 1). No animals were killed for the purposes of this study. Samples were collected from animals that died on different dates or locations, except for two pairs (an adult female and adult male, and two juvenile males). Therefore, sampling is unlikely to be biased towards related individuals. Sampling permits were issued by the Brazilian Environmental Agencies IBAMA/MMA (Instituto Brasileiro do Meio Ambiente e Recursos Renováveis; sampling permits 11495-1, 11980-1 and 25269-1) and ICMBio/MMA (Instituto Chico Mendes de Conservação da Biodiversidade; sampling permits 11579-1 and 20264-5). DNA was isolated through the standard phenol-chloroform procedure with proteinase K [31]. We used the complete mitochondrial genome of *Pontoporia blainvillei* (GenBank NC005277) to design a new set of primers, flanking 577 base pairs (bp) of the mitochondrial control region, (RCPb-F 5′- CTC CTA AAT TGA AGA GTC TTC G – 3′; RCPb-R 5′ – CCA TCG AGA TGT CTT ATT TAA GAG G – 3′). PCR amplification was performed in 25 µL reactions containing 1 unit of GoTaq polymerase (Promega); 0.20 mM dNTPs; 2.5 mM MgCl2; 25 µg BSA and 0.5 µM of each primer. PCR cycling was as follows: 3 min. at 93°C; 30 cycles of 1 min. at 92°C, 1 min. at 50°C and 1 min. at 72°C; plus 5 min. of final extension at 72°C. PCR products were purified and sequenced in both directions in an ABI 3130 automated sequencer. Sequences were edited with program *SeqMan 7* (Lasergene Inc.), visually aligned in *MEGA 4* [32] and submitted to GenBank, under accession numbers KF270687 to KF270692.

Previously published sequences from different localities (N = 94 [27], [33]) were included in the alignment, increasing sample size to 162 and covering the species' entire range (Fig. 1). The two sampling sites from Rio de Janeiro (RJS and RJN) came from different sides of a gap in the current distribution of the species.

Haplotype and nucleotide diversities were estimated with *DNASp 5* [34]. Population differentiation analyses (AMOVA [35]) were conducted in *Arlequin 3.5* [36]. Mismatch distribution analyses and a Mantel test were also performed in *Arlequin 3.5*. A median joining haplotype network was built with *Network 4.611* ([37], www.fluxus-engineering.com).

We investigated the demographic past of the species with a Bayesian skyline plot reconstruction conducted in *BEAST 1.6* [38]. Coalescent reconstructions used a strict molecular clock with the mutation rate for the control region of cetaceans (estimated at 1%/My [39]) and the HKY + I mutation model, as indicated by *jModelTest* [40]. The number of grouped intervals (m) was set to five. Three independent runs of ten million Markov Chain Monte Carlo (MCMC) steps each were performed to achieve reliable parameters estimates (ESS>200).

## Results

**Figure 2:**
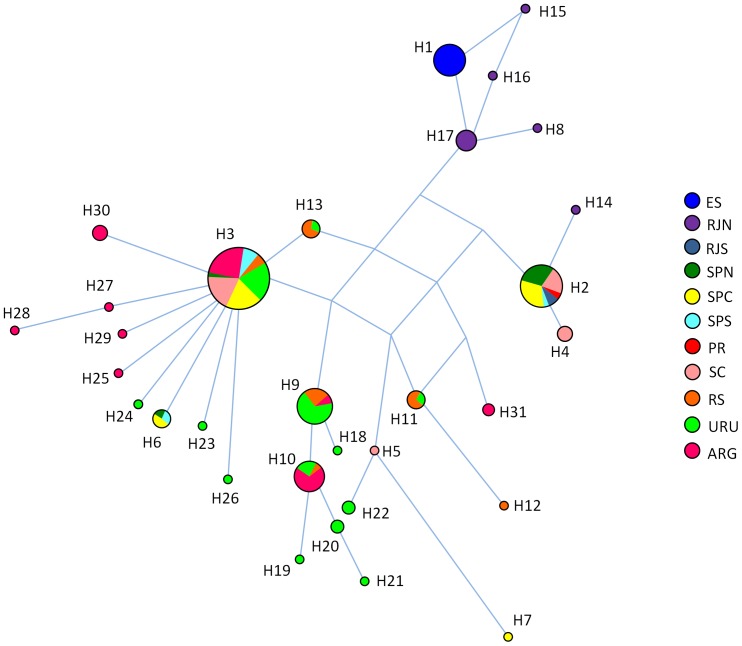
Median-joining network of franciscana control region haplotypes. Relationship among 30 haplotypes determined by analysis of 455

Due to the shorter length of published sequences, analyses were conducted using an alignment of 455 bp. Thirty-six substitutions were observed, defining 30 haplotypes, of which six had not been reported previously. Haplotype and nucleotide diversities were 0.868 (±0.018) and 0.009 (±0.00035), respectively. A gradient of haplotype diversity was evident, decreasing from south to north, and all samples from the northernmost sampling area (Espírito Santo) shared the same, exclusive haplotype (Fig. 2, Table S1 and Figure S1).

The most frequent haplotype (H3) was found in all localities south of SPC, and the second most common haplotype (H2) occurred in all localities between SC and RJS (Fig. 2). Haplotype H3 is connected to many other haplotypes, forming a star-shaped topology suggestive of population expansion. Haplotypes from RJN and ES are closely related, but their connection to haplotypes from other localities could not be precisely defined. Haplotype H14 (haplotype E from [33]) was observed in a single individual from the north, but it groups with haplotypes from the south. Since confirmation of that sequence was not made and is not feasible at present (ER Secchi, personal communication), we decided to remove H14 from the analyses.

All biologically plausible groupings of geographically adjacent populations, varying the number of populations (K) from two to seven, were tested using the AMOVA framework (Table S2). The population structure hypotheses tested included those previously proposed ([e.g. 33], [41]). Considering all localities, AMOVA gave stronger support (Ф_CT_ = 0.44; P<10^−5^) to a two-population scenario (AR+UR+RS+SC+PR+SP+RJS/RJN+ES; Table 1, Table S2). Overall population structuring was also observed when the highly differentiated samples from RJN and ES were excluded (Ф_ST_ = 0.19; P<10^−5^). Thus, sub-structuring was further investigated among all localities south of RJS (Table S3). The most likely AMOVA scenario was of three populations (Ф_CT_ = 0.20; P<10^−5^): ARG+URU+RS/SC+PR+SPS+SPC/SPN+RJS (Table 1). Similarly, the separate analysis of RJN and ES also revealed population subdivision in the northern part of the distribution (Ф_ST_ = 0.72; P<10^−5^) (Table 1, Table S4). Combining all results, our analyses indicate the existence of five franciscana populations (ARG+URU+RS/SC+PR+SPS+SPC/SPN+RJS/RJN/ES), of which RJN and ES are the genetically most differentiated. The existence of isolation by distance in the species was not supported by the Mantel test (P = 0.69, Figure S2).

**Table 1:** Detailed AMOVA results of the most likely population structure scenarios including all localities (a) and excluding ES and RJN (b), and of the rejected scenarios of panmixia in the northern (c) and southern (d) parts of the species' range.

	Sum of squares	Variance components	Percentage variation	Ф Statistics	P
a) 2 populations, all localities: ARG+URU+RS+SC+PR+SP+RJS/RJN+ES					
Among groups	80.607	1.74510	42.21651	0.44(Ф_CT_)	10^−5^
Among populations/within groups	81.996	0.52228	12.63473		
Within populations	281.211	1.86631	45.14876		
b) 3 populations, without ES and RJN: AR+UR+RS/SC+PR+SPS+SPC/SPN+RJS					
Among groups	48.861	0.56088	19.97636	0.20 (Ф_CT_)	10^−5^
Among populations/within groups	24.676	0.14415	5.13397		
Within populations	270.711	2.10267	74.88967		
c) Single northern population, RJN+ES					
Among populations	8.458	0.68409	58.90411	0.72 (Ф_ST_)	10^−5^
Within populations	10.500	0.47727	41.09589		
d) Single southern population, ARG+URU+RS+SC+PR+SP+RJS					
Among populations	73.538	0.50129	19.25106	0.19 (Ф_ST_)	10^−5^
Within populations	270.711	2.10267	80.74894		

The population groups detected by AMOVA analyses were evaluated in relation to possible population expansions, and all of them (except ES, which could not be analysed) had mismatch distributions compatible with the sudden population and geographic expansion models (Figure S3). Expansions were dated from around one million years before present (ybp; SPN+RJS and SC+PR+SPS+SPC) to less than 100,000 years ago (RJN) (Figure S3).

The Bayesian skyline plots revealed contrasting demographic histories among the four populations analysed (Figure S4). Very recent demographic trends cannot be determined due to the stochasticity of the coalescent process, which results in large variances [38], but older patterns can be more clearly depicted. The population from RJN may have had a slight increase for the past 125,000. Population SPN+RJS showed stable population size during the last 250,000 years. Estimates from those two populations had larger variances also as a consequence of smaller sample sizes. Population SC+PR+SPS+SPC seems to have experienced a steady decline which began around 100,000 years ago. Population ARG+URU+RS would have begun expanding 250,000 years ago, with a steeper increase 50,000 years ago. Demographic trends should be regarded as preliminary, because they were based on a single *locus* [42].

## Discussion

This is the geographically most comprehensive study on the genetic structure and molecular demography of franciscanas to date. The analyses reveal that the species is subdivided into two Evolutionarily Significant Units, each with a higher number of populations (Franciscana Management Areas) than previously recognised. The corollary is that the four current FMAs are inadequate to ensure the best protection for all populations, thus prompting the need for reassessing FMAs.

### Population structure

This is the first study to analyse genetic samples from the northernmost population of *Pontoporia*. Interestingly, our results unequivocally show that samples from that area (ES) and those from northern Rio de Janeiro (RJN) comprise populations that are different from each other and much differentiated from those southwards along the South-American coast. Franciscanas from those two areas were provisionally pooled in Franciscana Management Area I (FMAI, [26]), acknowledging the lack of biology and genetic data for the area. However, franciscanas from ES, RJN and SP have been shown to have non-overlapping craniometrical measures [43]. Recently, significant differences were reported in the external morphology of franciscanas from FMAI (RJN only), FMAII and FMAIII [44]. Combining those results with previous data on genetics, growth, demography and reproduction, Barbato et al. [44] suggested that RJN could be an Evolutionarily Significant Unit (ESU, *sensu* Ryder [45]).

**Figure 3:**
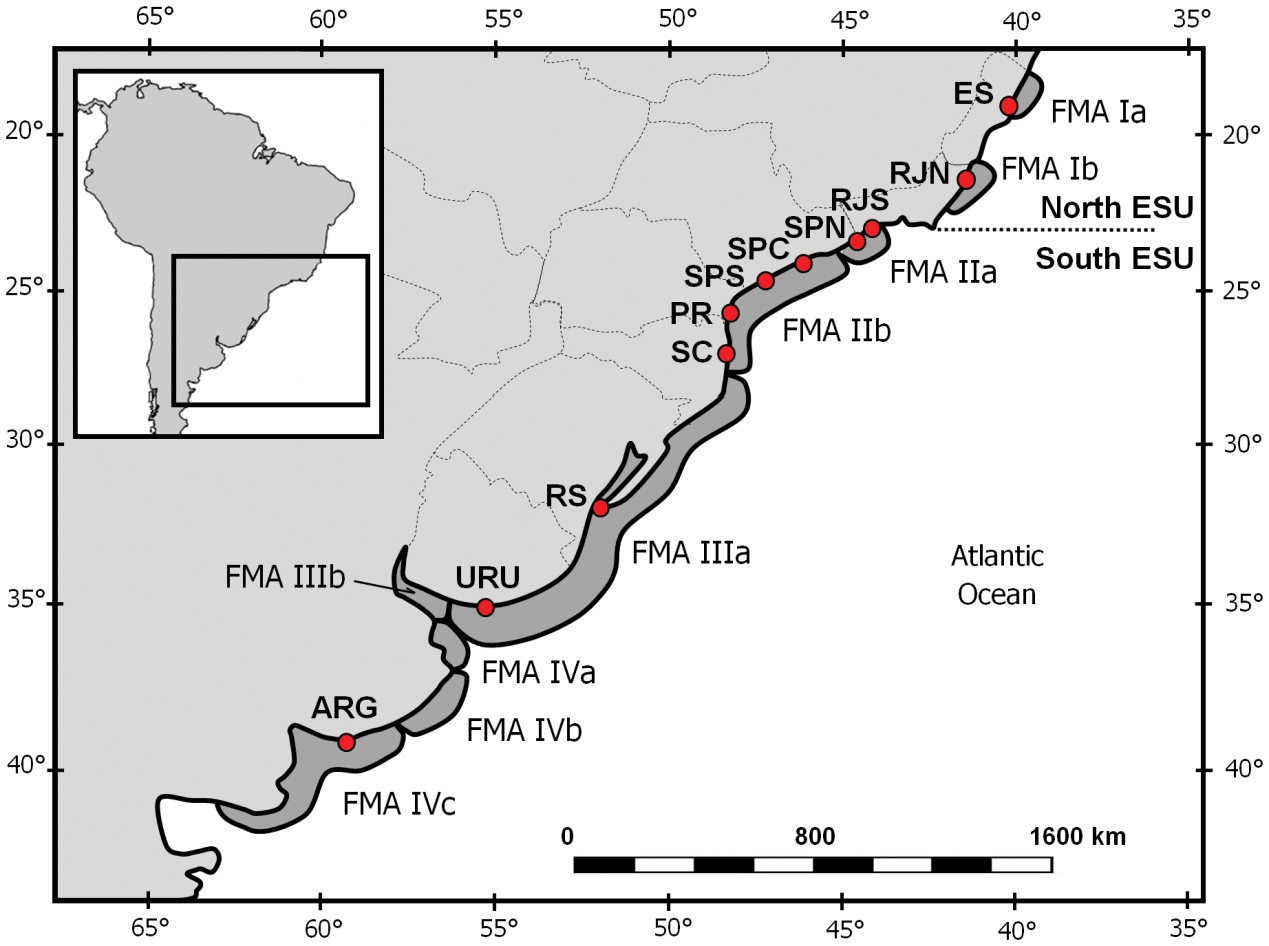
Reassessment of the FMA proposal of Secchi et al. (2003) according to the present analyses. A deep evolutionary break separates franciscanas from North (ES, RJN) and South (RJS to ARG), justifying the recognition of two Evolutionarily Significant Units (ESU). Evidence of genetic differentiation further supports dividing the former FMAI and FMAII. The current proposal includes the subdivision of FMAIII and FMAIV, as suggested by Mendez et al. (2010) and Costa-Urrutia et al. (2012). See text for details.

Here, we provide clear evidence that franciscanas should, indeed, be divided into two ESU, North (ES and RJN) and South (RJS to ARG) (Fig. 3). The concept of ESU was operationally defined by Moritz [25] as a group of individuals showing reciprocal monophyly of DNA lineages. That condition is met by franciscana sequences from North and South when H14 is conservatively removed from analyses. Besides fulfilling the qualitative *criteria* of Moritz [25], North and South are also quantitatively much differentiated (Φ_CT_ = 0.42 or 0.44, with or without H14, respectively).

Our results also reveal that the North ESU should be split into two Management Units (*sensu* Moritz [25]). For the sake of coherence with the current classification scheme, they will be termed, here, FMAIa (ES) and FMAIb (RJN). However, the highly differentiated status of the North ESU as a whole must not be downplayed (Fig. 3).

The original range of FMAI included the two gaps in the species distribution. This study analysed for the first time genetic samples from within those two gaps. Those samples allowed a more precise delimitation of Management Units and ESUs (Fig. 3). One sample came from the northernmost gap and belonged to population ES (FMAIa), extending its southern limit to Santa Cruz (19°56'S). The two samples from RJS were collected inside the other gap and grouped with SPN, confirming that that population extends further north than previously thought, as already suggested by Azevedo et al. [46].

The scenario of five populations (AR+UR+RS/SC+PR+SPS+SPC/SPN+RJS/RJN/ES) supported by AMOVA, is at odds with the FMA proposal of Secchi et al. [26] not only because of the distinctiveness of ES and RJN, but also due to an additional subdivision within FMAII, which was also not found by Ott [47]. As we had samples from across the entire coast of the state of São Paulo, they were split into three localities, thus enabling the test of scenarios where they were part of the same or distinct populations. The most likely AMOVA scenario indicates that FMAII should encompass two Management Units, one including SPN+RJS (FMAIIa) and the other SPC to SC (FMAIIb) (Fig. 3). That conclusion is compatible with contaminants levels, which indicate heterogeneity among franciscanas from SP. Lailson-Brito et al. [20] analysed organochlorine loads and observed differences in ΣDDT/ΣPCB between SPN and SPS, but SPC was closer to SPN, while SPS was more similar to PR. It is important to note that some alternative scenarios had Φ_CT_ values only slightly lower (Table S3), so the subdivision of FMAII should be regarded as provisional and deserves further scrutiny, using more samples and markers with higher resolution (like microsatellites).

Still concerning FMAII, our results differ from those of Ott [47], who also analysed samples from localities between RJN to URU (except for RJS), both because he did not find genetic differentiation within FMAII, but also because he suggested that southern SC was genetically closer to FMAIII than to FMAII. However, the apparent contradiction between this study and his is an artifact of sampling, because all SC samples that we studied came from the north of the state of Santa Catarina, while Ott [47] used samples from southern Santa Catarina. The existence of genetic differentiation within the state of Santa Catarina was later indicated by a preliminary study using 13 samples [48]. Thus, combining our results and those of Ott [47] and Ott et al. [48], the limit between FMAIIb and FMAIII would lie somewhere at the center of the coast of the state of Santa Catarina (Fig. 3).

Franciscana populations from both sides of the La Plata River have been treated as different Management Units (FMAIII and IV) based on infection levels and diet composition [26]. This differentiation is further supported by analyses of external morphology [44] and of control region haplotype frequencies [27], [28]. Contrastingly, sequence-based analyses of the control region failed to detect differences between the two areas [27], [28]. In this study, FMA III and FMA IV could not be discriminated by AMOVA of control region sequences, as scenarios that separated them had consistently low or non-significant Φ_CT_ values. However, we believe that those Management Units should be maintained based on the precautionary approach, since previous studies with microsatellite data report small scale genetic differentiation within FMA III and IV [29], [30], and especially because franciscanas in that region must be managed by three different countries.

Recently, mtDNA and microsatellite data indicated geographic micro-scale differentiation among localities within FMA IV [29], as previously suggested by preliminary mtDNA data [27], [28]. Microsatellite data also revealed fine-scale differentiation between franciscanas from the La Plata River and adjacent coastal waters [30]. The degree of differentiation among those local populations is very small compared to the high divergence observed among current FMAs, so studies encompassing the species as a whole (or even considering only sequences from across the South ESU) do not detect such micro-geographic genetic differences ([e.g. 47], this study).

Micro-scale genetic differentiation is highly relevant to the management of franciscanas, and as such should be investigated across the entire range of the species. The goal of conservation biology is to preserve species in space and time, and that latter axis depends on maintaining the evolutionary potential contained in geographically restricted adaptive variation. Those local populations should be managed independently on a local basis, to avoid the loss of such adaptations. Therefore, we propose that FMA III and IV should also be updated to accommodate micro-scale genetic differentiation, as suggested by previous studies ([27], [28], [29], [30] Fig. 3). The implementation of marine protected areas may be the best way to ensure the viability of local populations.

Although microsatellite data will be helpful to address micro-scale genetic structure in franciscanas, major patterns of differentiation, as obtained through mitochondrial data, should not change. That conclusion is supported by previous studies that analysed mitochondrial and microsatellite data and observed concordant population structure across markers [28], [29], [47].

### Demographic trends

Combining the results of both demographic analyses (mismatch analyses and Bayesian skyline plots) we concluded that the ARG+URU+RS and the RJN populations were the only ones to experience demographic expansions in the recent past (around 250,000 and 100,000 ybp, respectively). Stable population sizes seem to have been kept by SPN+RJS (last 250,000 years), and SC+PR+SPS+SPC seems to have suffered a decline from around 100,000 years ago. Older demographic expansions appear to have occurred in all populations, possibly coupled with spatial expansions, as indicated by mismatch analyses. Although Bayesian skyline plots have large variances, it is possible to infer that the RS+URU+ARG population has kept a larger size than the other populations, even before the last demographic expansion. That seems to support the hypothesis that the colonization of the Southwestern Atlantic happened from the south northwards, as already proposed [3]. Franciscanas would have been in the area around the La Plata River for longer than anywhere, explaining their higher genetic diversity there.

### Relevance to management and conservation

Our results are very relevant to franciscanas' management, by reformulating the FMA proposal of Secchi et al. [26], currently adopted in all conservation plans for the species (e.g. the Brazilian Action Plan for the Conservation of Franciscanas [49]). The main conclusion of this study is the splitting of franciscanas into two Evolutionarily Significant Units, the North and South ESUs. In addition, our data show that both ESU should be further divided to reflect genetic differentiation. The North ESU comprises two FMAs, each in urgent need of specific research and conservation efforts. FMAIa (ES) is the least studied of all FMAs. Although there is no information on its abundance, ES may be a small population, as indicated by the relative low number of incidental captures [7], [50], [51], few sightings during an aerial survey [52] and extremely low genetic diversity (h = 0; N = 14; Table S1). As stated above, the goal of species conservation is to maintain them in time and space, so the loss of peripheral populations represents both a direct failure (of keeping the original geographical range) and an indirect threat to the species' long term persistence (by the possible reduction of adaptive potential). It is imperative to gather basic data on *Pontoporia* demography and life history, as well as on human-related mortality, so that the conservation status of ES (FMAIa) can be evaluated before its maintenance is irreversibly jeopardised. RJN (FMAIb), on the other hand, is a relatively well known population, but there is no data on its abundance. Still, this population has suffered substantial removal through bycatch, of around 110 animals each year [12]. The low level of genetic diversity supports the notion that ES, RJN and RJS+SPN populations are the smallest and most vulnerable.

The genetic discontinuity within FMAII warrants further investigation. Due to the fact that ecotoxicological data [20] seem to support such differentiation, we suggest that FMAII be provisionally split into two FMAs (FMA IIa and FMA IIb). Those two new FMAs appear to be relatively small, especially SPN+RJS, and inhabit a region under heavy human occupation. Thus, much of their original habitat has been lost or degraded by anthropogenic activities, while bycatch is also substantial [11], [53]. The analysis of microsatellite data and a larger sample size should clarify the existence of differentiation within FMAII.

Our results do not give support to the existence of more than a single genetic population from RS to ARG. However, we believe that FMAIII and FMAIV should be managed independently, irrespective of their low genetic differentiation. Those populations are the most studied in all aspects, including abundance, population parameters and fishery-related mortality [5], [15], [54], [55], [56], [57], [58], [59]. The high quality data acquired to date have enabled the analysis on the population viability of franciscanas [14], and granted the species a “vulnerable” conservation status [17]. Besides, the micro-geographic differentiation recently documented in Argentina (FMAIV) [28], [29] and Uruguay (FMAIII) [30] emphasises the need of preserving such local populations and others still to be discovered, as they possibly harbour exclusive adaptive variation. We urge that similar data be gathered for all other FMAs, especially of the North ESU, which may be even more vulnerable due to probably lower abundances. It is important to note that incidental captures may not be the greatest threat to franciscanas from SC northwards, which encompasses half of the species' distribution.

## Supporting Information

Figure S1 **Gradient of genetic diversity across the franciscana's geographic range.** Square: haplotype diversity; circle: nucleotide diversity.
(TIF)

Figure S2 **Mantel test based on control region sequences (N = 162).** The x axis is geographic distance (in km) and the y axis is the genetic distance (Rousset's linear F_ST_).
(TIF)

Figure S3 **Mismatch distributions of franciscana populations.** a) Sudden demographic expansion model, and b) spatial expansion model. Bars show the observed distribution and the line shows the expected distribution. Observed distributions were not statistically different from those expected under expansion models, as indicated by P values of the sum of squared deviations. “T” indicates time since expansion events, in years.
(TIF)

Figure S4 **Bayesian skyline plots (m = 5).** Derived from franciscana mtDNA control region sequences from four populations: RJN (N = 9), SPN+RJS (N = 10), SC+PR+SPS+SPC (N = 44) and ARG+URU+RS (N = 84). The x axis is in years, and the y axis is equal to Neτ (the product of the effective population size and the generation length in years). The thick solid line is the mean estimate, and the grey area show the 95% highest posterior density (HPD) limits. Estimated times to most recent common ancestor (TMRCA) of the populations, in years, are indicated.
(TIF)

Table S1 **Genetic diversity in the mtDNA control region of franciscanas.** N: sample size; n: number of haplotypes; h: haplotype diversity; π: nucleotide diversity.
(PDF)

Table S2 AMOVA results of all population structure scenarios tested, considering all sampling localities, compared to scenarios proposed previously.
(PDF)

Table S3 AMOVA results of all population structure scenarios tested, excluding RJN and ES.
(PDF)

Table S4 AMOVA results for scenarios of panmixia.
(PDF)
