# Supplementary figures and images for: Population Structure of the Endangered Franciscana Dolphin (Pontoporia blainvillei): Reassessing Management Units
Source: PLoS One. 2014 Jan 31;9(1):e85633. doi: 10.1371/journal.pone.0085633 (PMC3908959; doi:10.1371/journal.pone.0085633)

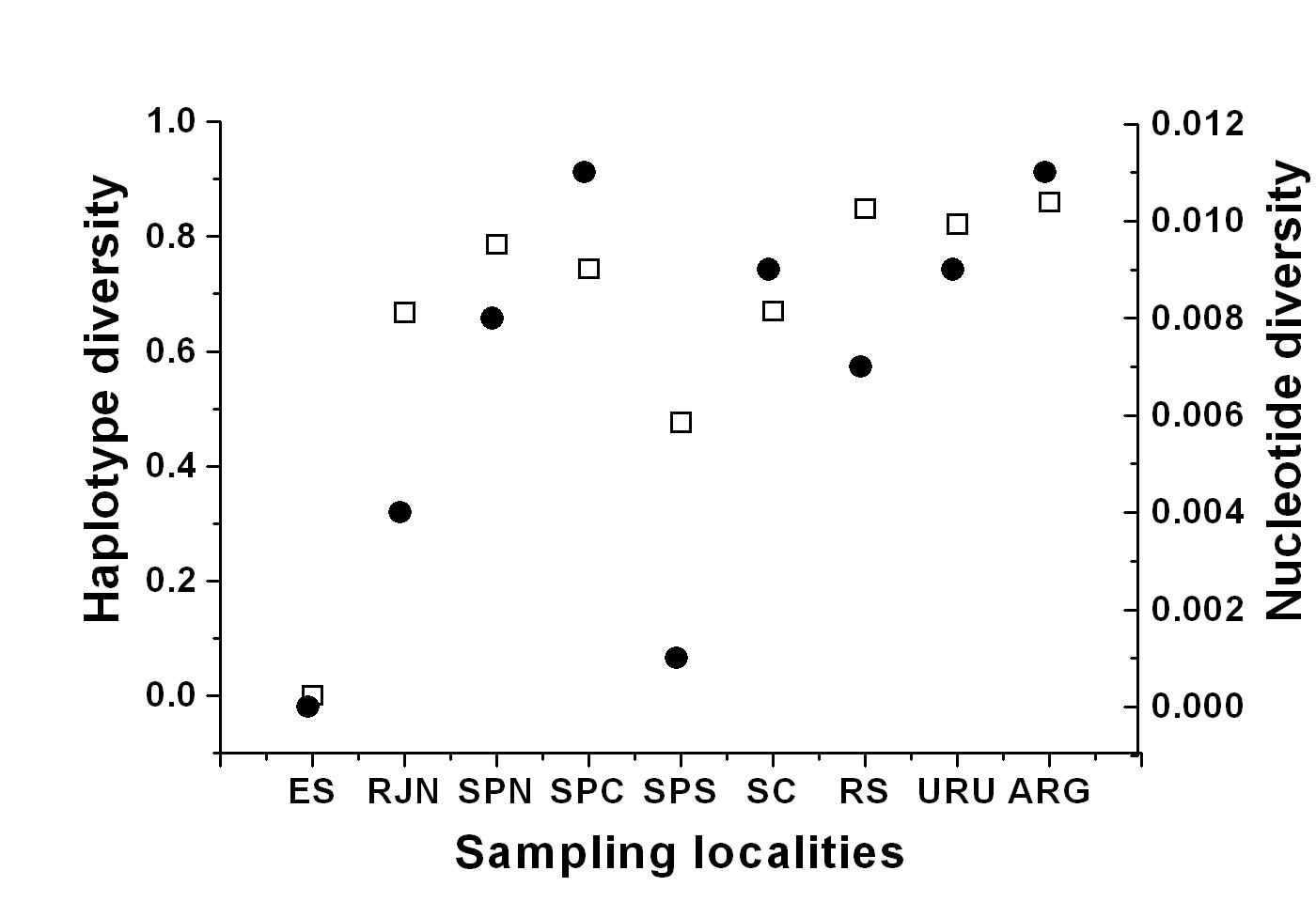

Supplement: Figure S1 — Gradient of genetic diversity across the franciscana's geographic range. Square: haplotype diversity; circle: nucleotide diversity. (TIF) [file pone.0085633.s001.tif]

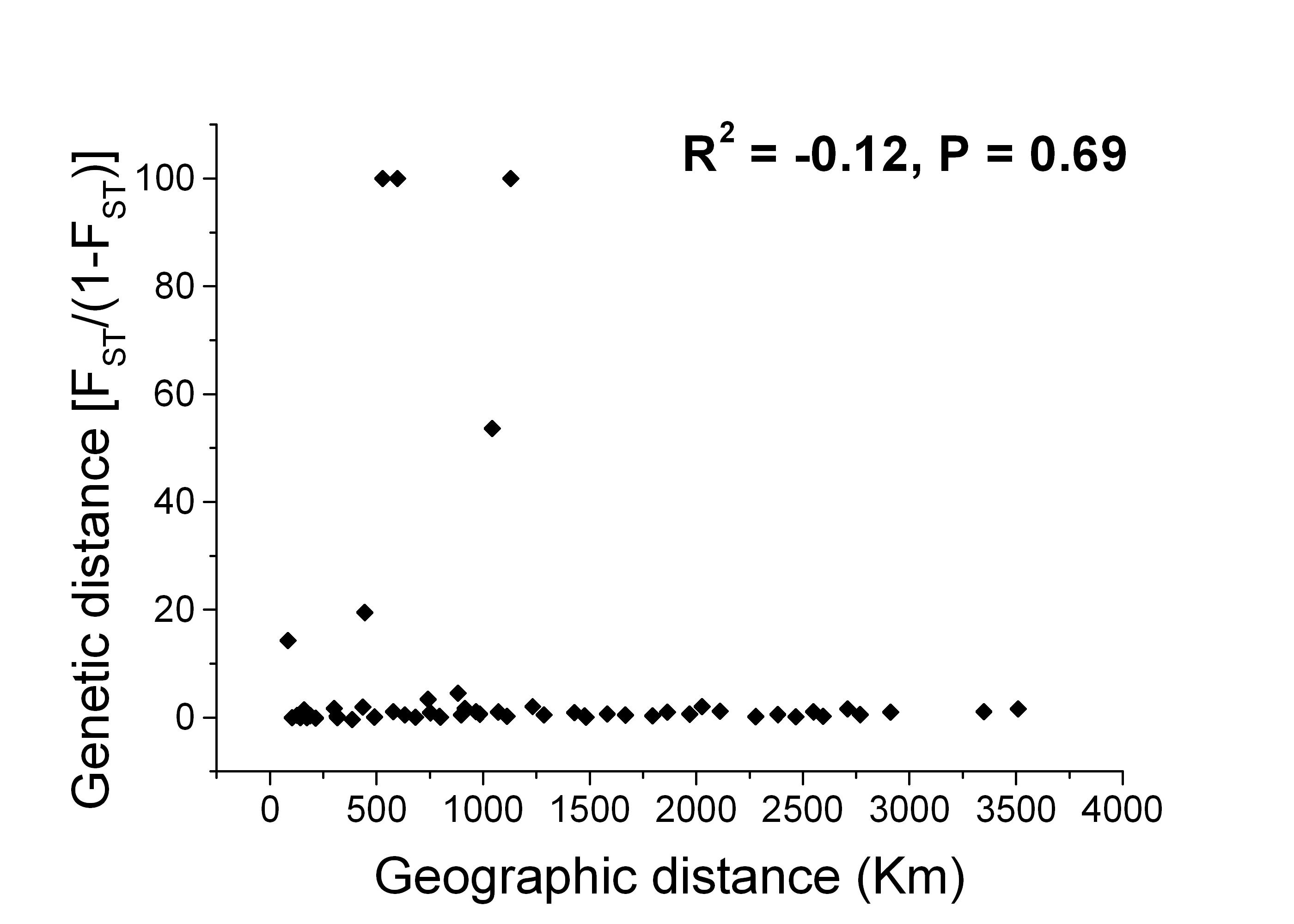

Supplement: Figure S2 — Mantel test based on control region sequences (N = 162). The x axis is geographic distance (in km) and the y axis is the genetic distance (Rousset's linear FST). (TIF) [file pone.0085633.s002.tif]

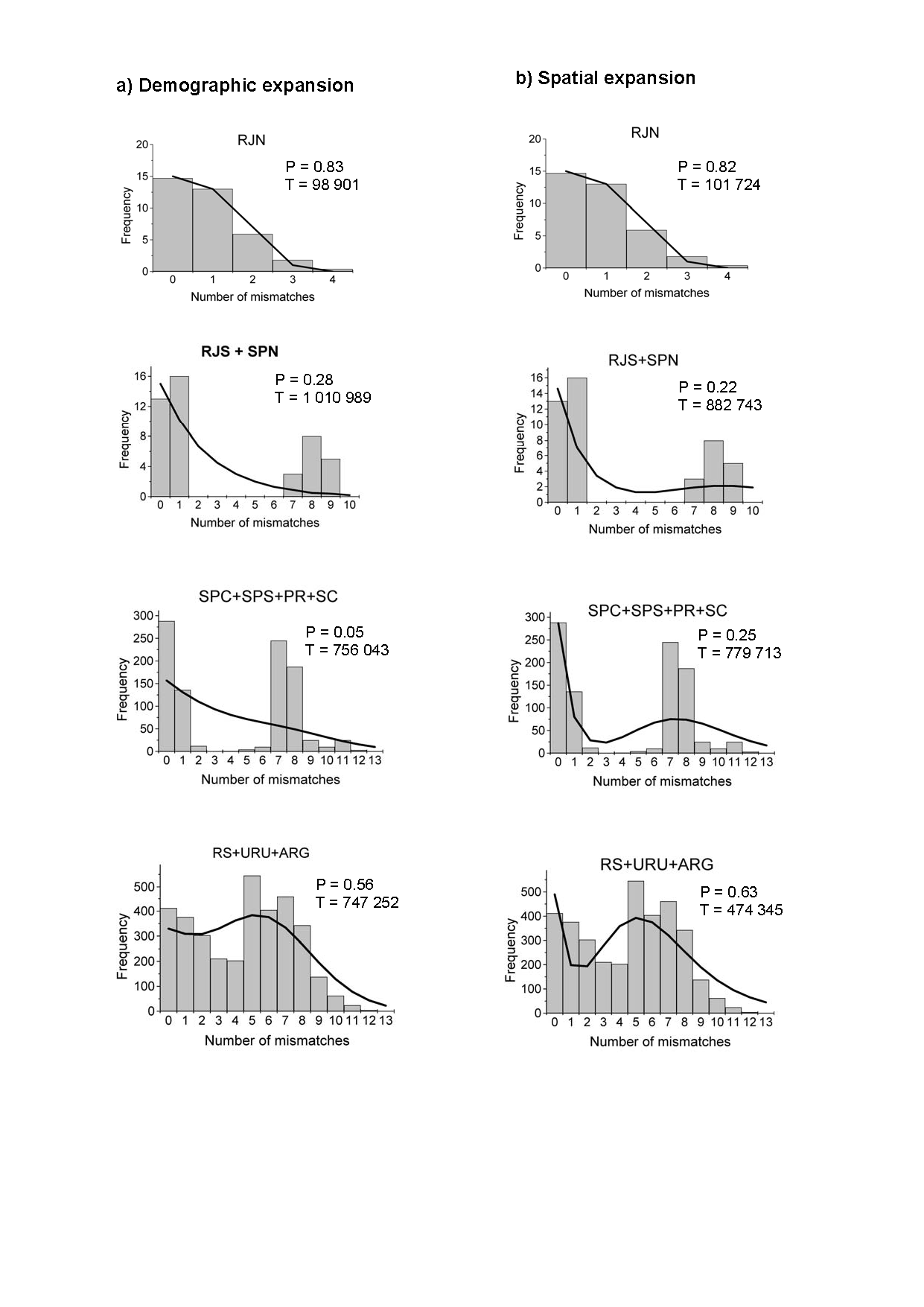

Supplement: Figure S3 — Mismatch distributions of franciscana populations. a) Sudden demographic expansion model, and b) spatial expansion model. Bars show the observed distribution and the line shows the expected distribution. Observed distributions were not statistically different from those expected under expansion models, as indicated by P values of the sum of squared deviations. “T” indicates time since expansion events, in years. (TIF) [file pone.0085633.s003.tif]

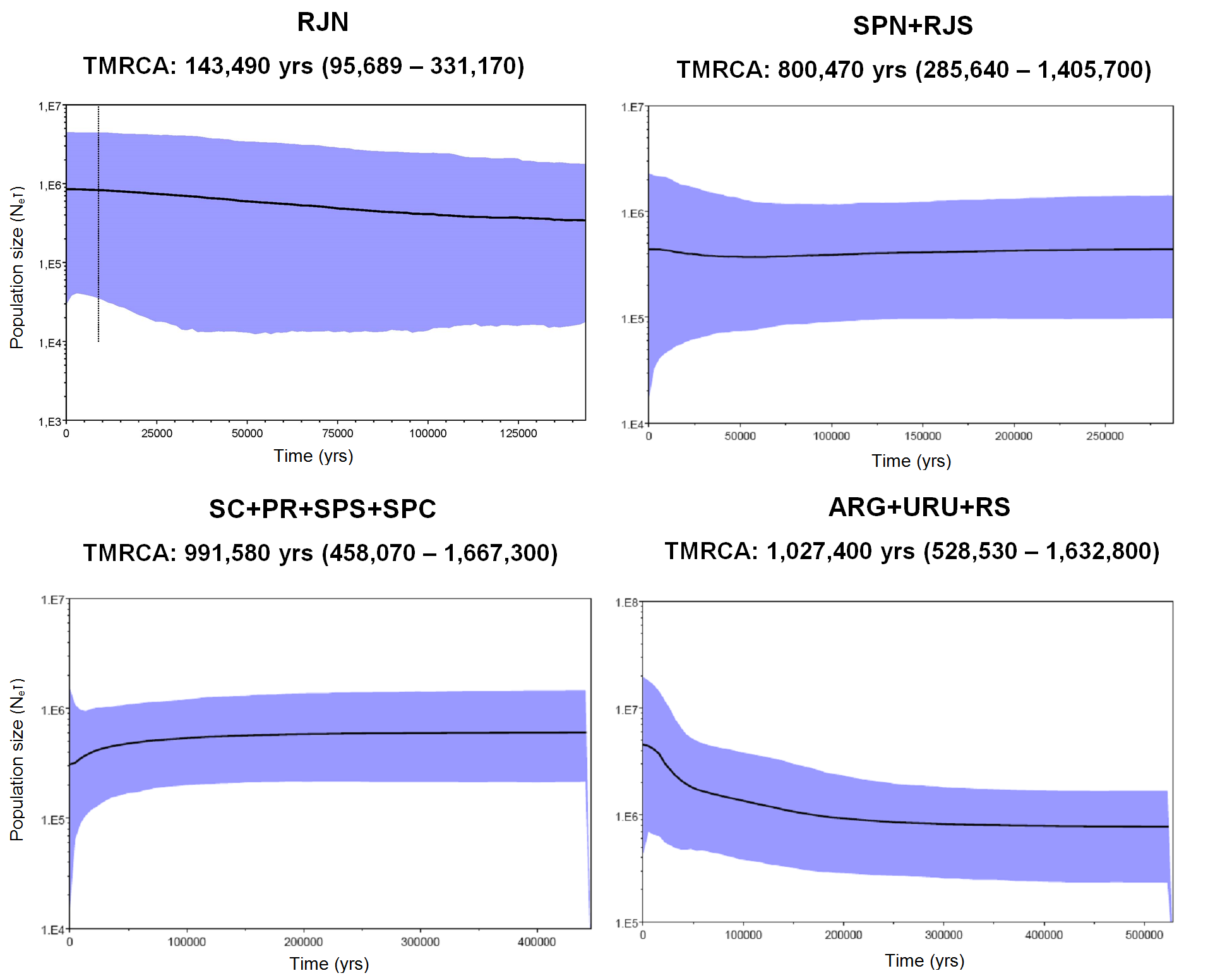

Supplement: Figure S4 — Bayesian skyline plots (m = 5). Derived from franciscana mtDNA control region sequences from four populations: RJN (N = 9), SPN+RJS (N = 10), SC+PR+SPS+SPC (N = 44) and ARG+URU+RS (N = 84). The x axis is in years, and the y axis is equal to Neτ (the product of the effective population size and the generation length in years). The thick solid line is the mean estimate, and the grey area show the 95% highest posterior density (HPD) limits. Estimated times to most recent common ancestor (TMRCA) of the populations, in years, are indicated. (TIF) [file pone.0085633.s004.tif]
