# Supplementary material for: Population Structure of the Endangered Franciscana Dolphin (Pontoporia blainvillei): Reassessing Management Units
Source: PLoS One. 2014 Jan 31;9(1):e85633. doi: 10.1371/journal.pone.0085633 (PMC3908959; doi:10.1371/journal.pone.0085633)
Supplement: Table S1 — Genetic diversity in the mtDNA control region of franciscanas. N: sample size; n: number of haplotypes; h: haplotype diversity; π: nucleotide diversity. (PDF) [file pone.0085633.s005.pdf]

Table S1: Genetic diversity in the mtDNA control region of franciscanas. N: sample size; n: number of haplotypes; h: haplotype diversity; π: nucleotide diversity.

Locality	***N***	***n***	***h***	***π***
Espírito Santo (ES)	14	1	0	0
Rio de Janeiro, North (RJN)	10	5	0.667	0.004
Rio de Janeiro, South (RJS)	2	1	0	0
São Paulo, North (SPN)	8	4	0.786	0.008
São Paulo, Central (SPC)	19	5	0.743	0.011
São Paulo, South (SPS)	7	2	0.476	0.001
Paraná (PR)	1	1	-	-
Santa Catarina (SC)	17	4	0.669	0.009
Rio Grande do Sul (RS)	15	6	0.848	0.007
Uruguay (URU)	38	13	0.821	0.009
Argentina (ARG)	31	11	0.860	0.011
