# Supplementary material for: Population Structure of the Endangered Franciscana Dolphin (Pontoporia blainvillei): Reassessing Management Units
Source: PLoS One. 2014 Jan 31;9(1):e85633. doi: 10.1371/journal.pone.0085633 (PMC3908959; doi:10.1371/journal.pone.0085633)
Supplement: Table S2 — AMOVA results of all population structure scenarios tested, considering all sampling localities, compared to scenarios proposed previously. (PDF) [file pone.0085633.s006.pdf]

Table S2: AMOVA results of all population structure scenarios tested, considering all sampling localities, compared to scenarios proposed previously.

Population structure hypotheses	Φ_{CT}	P
2 populations		
AR+UR+RS+SC / PR+SP+RJ+ES	0.13	0.004
AR+UR+RS / SC+PR+SP+RJ+ES [a]	0.13	0.02
AR+UR+RS+SC+PR+SP+RJS / RJN+ES	**0.44**	10^{-5}
AR+UR+RS+SC+PR+SP / RJ+ES	0.40	10^{-5}
3 populations		
AR+UR+RS+SC / PR+SP+RJS / RJN+ES	0.31	10^{-5}
AR+UR+RS / SC+PR+SP+RJS / RJN+ES	0.34	10^{-5}
AR+UR+RS+SC+PR+SP+RJS / RJN / ES	0.40	10^{-5}
AR+UR+RS+SC+PR+SP / RJ / ES	0.40	10^{-5}
AR+UR+RS+SC+PR+SPS+SPC / SPN+RJS / RJN+ES	0.41	10^{-5}
AR+UR+RS / SC+PR+SP+RJ / ES	0.25	10^{-5}
4 populations		
AR / UR+RS / SC+PR+SP / RJ+ES [b]	0.26	10^{-5}
AR / UR+RS / SC+PR+SP+RJS / RJN+ES	0.28	10^{-5}
AR+UR+RS / SC+PR+SP+RJS / RJN / ES	0.35	10^{-5}
AR+UR+RS+SC / PR+SP+RJS / RJN / ES	0.32	10^{-5}
AR / UR+RS / SC+PR+SP+RJ / ES	0.15	0.01
AR / UR+RS+SC / PR+SP+RJS / RJN+ES	0.22	10^{-5}
AR+UR+RS+SC+PR+SPS+SPC / SPN+RJS / RJN / ES	0.40	10^{-5}
5 populations		
AR / UR+RS / SC+PR+SP+RJS / RJN / ES	0.29	10^{-5}
AR+UR / RS+SC / PR+SP+RJS / RJN / ES	0.27	10^{-5}
AR / UR+RS+SC / PR+SP+RJS / RJN / ES	0.23	10^{-5}
AR / UR+RS / SC+PR / SP+RJS / RJN+ES	0.23	10^{-5}
AR+UR+RS / SC+PR+SPS+SPC / SPN+RJS / RJN / ES	0.36	10^{-5}
AR+UR+RS / SC+PR+SPS / SPC+SPN+RJS / RJN / ES	0.35	10^{-5}
AR+UR+RS+SC / PR+SPS+SPC / SPN+RJS / RJN / ES	0.33	10^{-5}
6 populations		
AR / UR+RS / SC+PR / SP+RJS / RJN / ES	0.24	10^{-5}
AR+UR+RS / SC+PR / SPS+SPC / SPN+RJS / RJN / ES	0.34	10^{-5}
AR / UR+RS / SC+PR+SPS+SPC / SPN+RJS / RJN / ES	0.32	10^{-5}
AR+UR+RS / SC / PR+SPS+SPC / SPN+RJS / RJN / ES	0.33	10^{-5}
AR+UR+RS / SC+PR / SPS / SPC+SPN+RJS / RJN / ES	0.37	10^{-5}
AR / UR+RS / SC+PR+SPS / SPC+SPN+RJS / RJN / ES	0.30	10^{-5}
7 populations		
AR / UR+RS / SC+PR / SPS+SPC / SPN+RJS / RJN / ES	0.28	10^{-5}

Population structure hypotheses proposed previously:
[a] Pinedo (1991) and Secchi *et al.* (1998).
[b] Secchi *et al.* (2003). See text for explanation.
