# Supplementary material for: Population Structure of the Endangered Franciscana Dolphin (Pontoporia blainvillei): Reassessing Management Units
Source: PLoS One. 2014 Jan 31;9(1):e85633. doi: 10.1371/journal.pone.0085633 (PMC3908959; doi:10.1371/journal.pone.0085633)
Supplement: Table S3 — AMOVA results of all population structure scenarios tested, excluding RJN and ES. (PDF) [file pone.0085633.s007.pdf]

Table S3: AMOVA results of **all** population structure scenarios tested, excluding RJN and ES.

**Population structure hypotheses (excluding RJN and ES)**	**Φ_{CT}**	**P**
2 populations		
AR+UR+RS / SC+PR+SP+RJS	0.18	0.0001
3 populations		
AR+UR+RS / SC+ PR / SP+RJS	0.13	10^{-5}
AR+UR+RS / SC+PR+SPS / SPC+SPN+RJS	0.17	10^{-5}
AR / UR+RS / SC+PR+SP+RJS	0.12	0.003
AR / UR+RS+SC+PR / SP+RJS	0.01	0.208
AR+UR+RS / SC+PR / SP+RJS	0.13	0.0001
AR+UR+RS / SC+PR+SPS+SPC / SPN+RJS	**0.20**	10^{-5}
4 populations		
AR / UR+RS / SC+PR+SPS+SPC / SPN+RJS	0.16	10^{-5}
AR / UR+RS / SC+PR / SP+RJS	0.06	0.02
AR+UR+RS / SC+PR / SPS+SPC / SPN+RJS	0.16	10^{-5}
AR+UR+RS+SC / PR+SPS / SPC / SPN+RJS	0.14	10^{-5}
AR+UR+RS / SC+ PR+SPS / SPC / SPN+RJS	0.17	10^{-5}
AR / UR+RS / SC+ PR+SPS / SPC+SPN+RJS	0.13	0.0001
5 populations		
AR / UR+RS / SC+PR / SPS+SPC / SPN+RJS	0.12	0.0005
AR / UR+RS / SC+PR+SPS / SPC / SPN+RJS	0.13	0.0004
AR+UR+RS / SC / PR+SPS / SPC / SPN+RJS	0.18	10^{-5}
6 populations		
AR / UR+RS / SC / PR+SPS / SPC / SPN+RJS	0.14	0.0005
7 populations		
AR / UR / RS / SC / PR+SPS / SPC / SPN+RJS	0.01	0.52
