# Supplementary material for: Population Structure of the Endangered Franciscana Dolphin (Pontoporia blainvillei): Reassessing Management Units
Source: PLoS One. 2014 Jan 31;9(1):e85633. doi: 10.1371/journal.pone.0085633 (PMC3908959; doi:10.1371/journal.pone.0085633)
Supplement: Table S4 — AMOVA results for scenarios of panmixia. (PDF) [file pone.0085633.s008.pdf]

Table S4: AMOVA results for scenarios of panmixia.

**Tests of population subdivision**	**Φ_{ST}**	**P**
Panmixia across the species' range	0.36	10^{-5}
South (AR + UR + RS + SC + PR + SP + RJS)	0.19	10^{-5}
North (RJN + ES)	0.72	10^{-5}

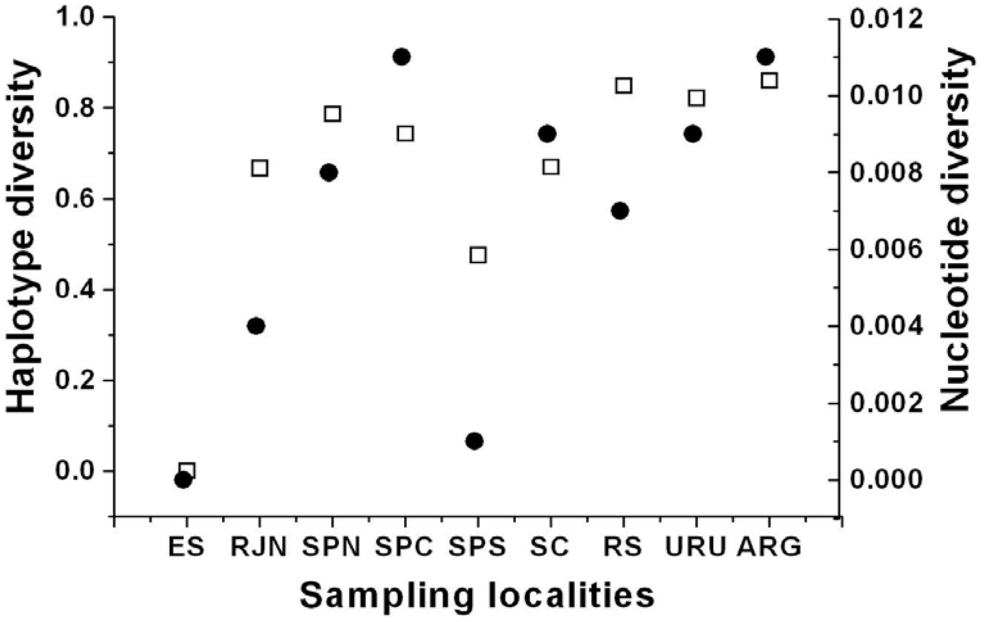

Figure A1: Gradient of genetic diversity across the franciscana's geographic range. Square: haplotype diversity; circle: nucleotide diversity.

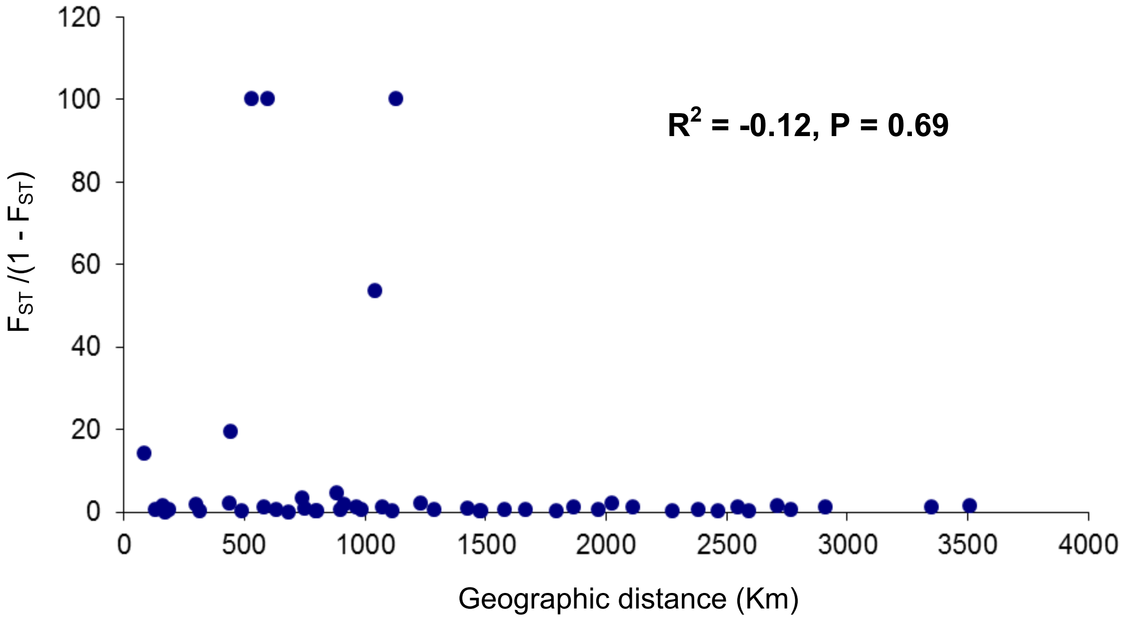

Figure A [illegible] egion sequ[illegible]
geograp[illegible] s is the ge[illegible]
F_{ST}).

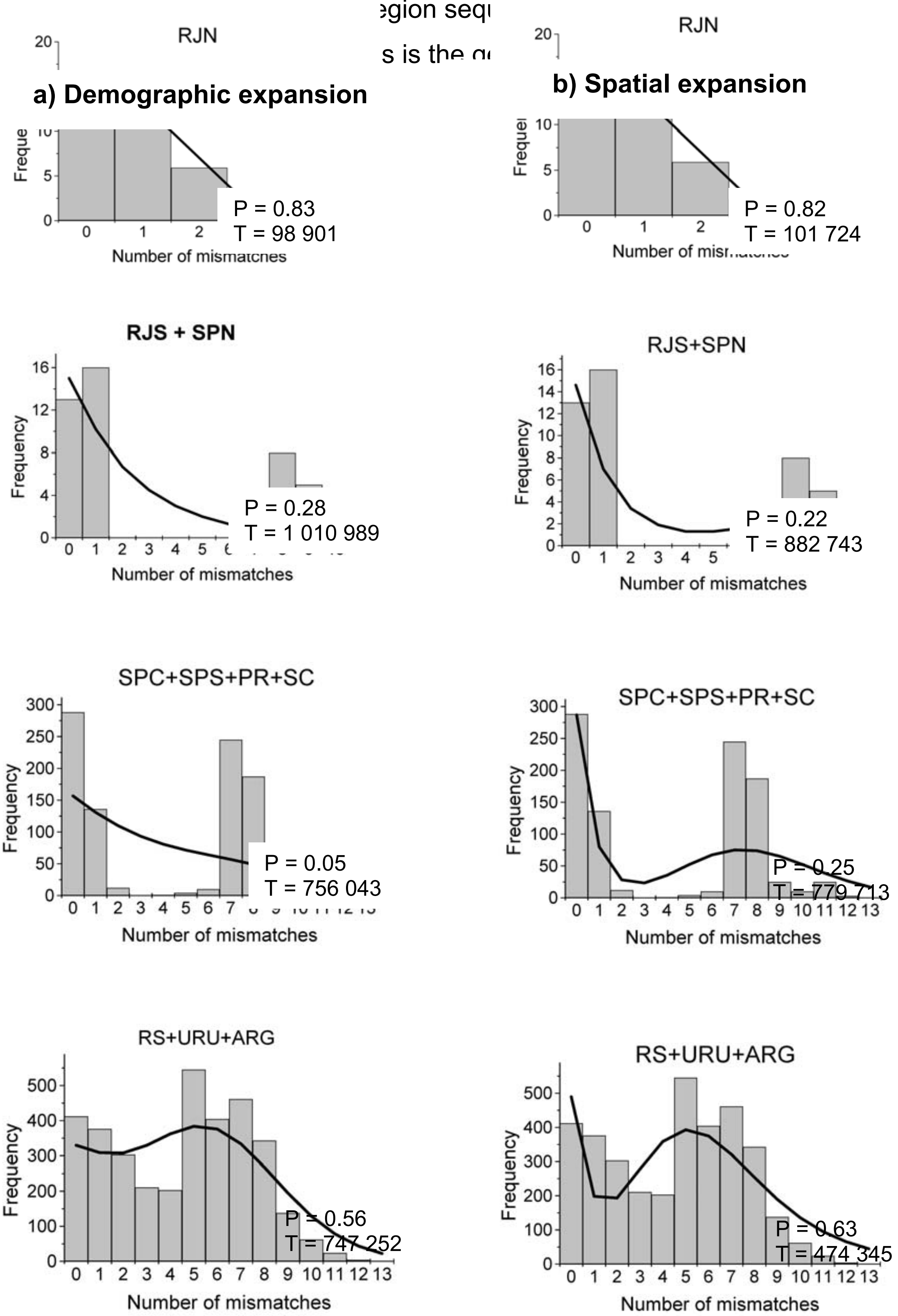

Figure A3: Mismatch distributions of franciscana populations according to the a) sudden demographic expansion model, and b) spatial expansion model. Bars show the observed distribution and the line shows the expected distribution. Observed distributions were not statistically different from those expected under expansion models, as indicated by P values of the sum of squared deviations. "T" indicates time since

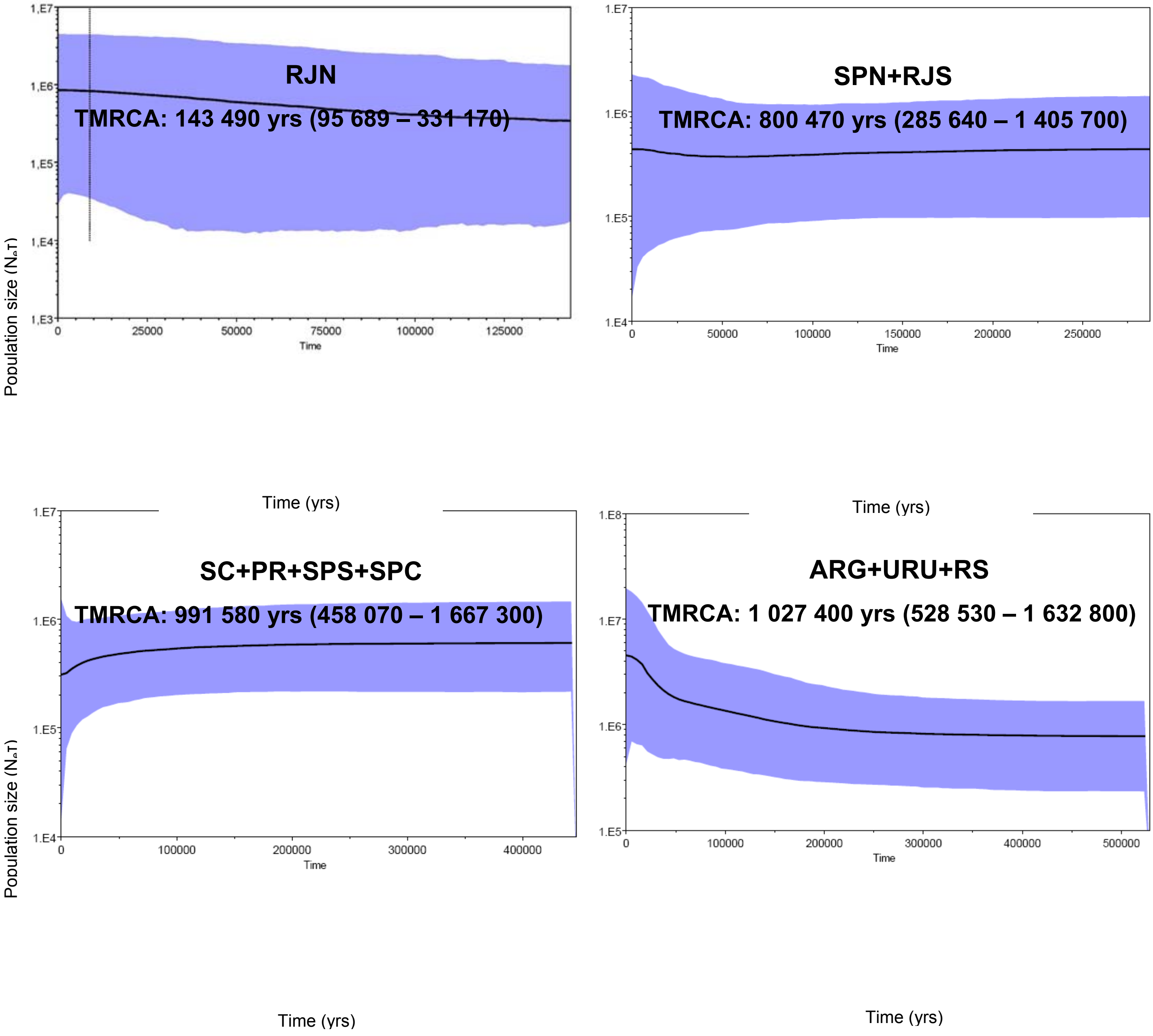

Figure A4: Bayesian skyline plots (*m* = 5) derived from franciscana mtDNA control region sequences from four populations: RJN (N = 9), SPN+RJS (N = 10), SC+PR+SPS+SPC (N = 44) and ARG+URU+RS (N = 84). The x axis is in years, and the y axis is equal to $N_e\tau$ (the product of the effective population size and the generation length in years). The thick solid line is the mean estimate, and the grey area show the 95% highest posterior density (HPD) limits. Estimated times to most recent common ancestor (TMRCA) of the populations, in years, are indicated.
